# A pilot study to assess residential noise exposure near natural gas compressor stations

**DOI:** 10.1371/journal.pone.0174310

**Published:** 2017-04-03

**Authors:** Meleah D. Boyle, Sutyajeet Soneja, Lesliam Quirós-Alcalá, Laura Dalemarre, Amy R. Sapkota, Thurka Sangaramoorthy, Sacoby Wilson, Donald Milton, Amir Sapkota

**Affiliations:** 1 Maryland Institute for Applied Environmental Health, School of Public Health, University of Maryland, College Park, Maryland, United States of America; 2 Department of Anthropology, College of Behavioral and Social Sciences, University of Maryland, College Park, Maryland, United States of America; North Carolina State University, UNITED STATES

## Abstract

**Background:**

U.S. natural gas production increased 40% from 2000 to 2015. This growth is largely related to technological advances in horizontal drilling and high-volume hydraulic fracturing. Environmental exposures upon impacted communities are a significant public health concern. Noise associated with natural gas compressor stations has been identified as a major concern for nearby residents, though limited studies exist.

**Objectives:**

We conducted a pilot study to characterize noise levels in 11 homes located in Doddridge County, West Virginia, and determined whether these levels differed based on time of day, indoors vs. outdoors, and proximity of homes to natural gas compressor stations. We also compared noise levels at increasing distances from compressor stations to available noise guidelines, and evaluated low frequency noise presence.

**Methods:**

We collected indoor and outdoor 24-hour measurements (L_eq, 24hr_) in eight homes located within 750 meters (m) of the nearest compressor station and three control homes located >1000m. We then evaluated how A-weighted decibel (dBA) exposure levels differed based on factors outlined above.

**Results:**

The geometric mean (GM) for 24-hour outdoor noise levels at homes located <300m (L_eq,24hr_: 60.3 dBA; geometric standard deviation (GSD): 1.0) from the nearest compressor station was nearly 9 dBA higher than control homes (L_eq,24hr_: 51.6 dBA; GSD: 1.1). GM for 24 hour indoor noise for homes <300m (L_eq,24hr_: 53.4 dBA; GSD: 1.2) from the nearest compressor station was 11.2 dBA higher than control homes (L_eq,24hr_: 42.2 dBA; GSD: 1.1). Indoor average daytime noise for homes <300m of the nearest compressor stations were 13.1 dBA higher than control homes, while indoor nighttime readings were 9.4 dBA higher.

**Conclusions:**

Findings indicate that living near a natural gas compressor station could potentially result in high environmental noise exposures. Larger studies are needed to confirm these findings and evaluate potential health impacts and protection measures.

## Introduction

In recent decades, there has been a sharp increase in unconventional natural gas development across the United States. From 2000 to 2015, natural gas production increased 40%, from 19.2 quadrillion British Thermal Units (BTU) to 27.0 quadrillion BTUs, and is expected to continue to increase to 33.1 BTUs by 2040 [1,2]. Much of this growth is related to technological advances in horizontal drilling and high-volume hydraulic fracturing that have allowed access to shale gas deposits. Production from shale gas deposits increased 2,588% from 2000 to 2013 and this trend is expected to continue [2]. A number of emerging studies have highlighted public health concerns regarding exposures to chemical, physical, and psychosocial hazards from unconventional natural gas development and production (UNGDP) and its impact on nearby communities [3–5].

Noise, or unwanted sound, is a physical hazard associated with UNGDP that has been identified as a major environmental health concern for nearby residents and communities [6–8]. Previous studies have linked chronic noise exposures in other settings to a wide variety of adverse effects. For example, long-term exposure to noise levels ranging from 32 to 75 A-weighted decibels (dBA) have been associated with sleep disruption, poor academic performance, and hypertension [9]. Other adverse health effects reported include noise-induced hearing loss, oxidative stress, increased cardiovascular effects, endocrine disruption, and an increased risk of developing diabetes [9,10]. There is also a growing concern that low frequency noise (10–250 Hz) can disrupt sleep, contribute to poorer performance (e.g., poor concentration and attention span), and cause annoyance [11,12]. The adverse health effects from noise are dependent on several factors, including duration, frequency, and intensity of exposure as well as individual physical and personal characteristics (e.g., age, pre-existing medical conditions, and intake of medications that are ototoxic) [13,14]. Children, elderly, and hearing impaired individuals may be more susceptible to the adverse effects associated with environmental noise exposures [13].

Recent reports indicate that noise levels associated with natural gas development, including truck traffic, well pad construction, and hydraulic fracturing are likely to be higher than 55 dBA [7,15,16], the U.S. Environmental Protection Agency’s (EPA) recommended outdoor noise limit to prevent activity interference and annoyance [17]. While increased noise levels are associated with both natural gas development and production, noise exposure associated with the development process is temporary. In contrast, exposures associated with the production and delivery of natural gas can be prolonged, impacting communities for extended periods of time. For example, compressor stations are permanent fixtures in communities where the production is active, and noise resulting from such facilities will continue to have an impact in the communities for decades to come [6,18]. Still, limited information exists regarding noise exposures associated with natural gas compressor stations and how they may impact nearby communities.

As part of a health impact assessment (HIA) on UNGDP in the Marcellus Shale in Western Maryland [6], we conducted a pilot study in West Virginia to assess whether compressor stations could pose a noise hazard and impact nearby community residents. In this study, we characterize compressor station related noise exposure at varying distances, locations (indoors vs. outdoors), and time of day (day vs. night) to help inform future studies and measures to protect public health [19].

## Methods

Noise monitoring was conducted around two natural gas compressor stations near a UNDGP site in Doddridge County, West Virginia between April 11^th^ to 17^th^, 2014, using 3M Quest SoundPro SE/DL series (3M Personal Safety Division, St. Paul, MN) sound level meters (SLMs), hereafter referred to as noise monitors. These noise monitors were used to assess area noise exposure levels. Compressor stations and participants were identified with the help of a local community group and were selected based on convenience. All monitors were set to collect slow response, A-weighted sound levels (dBA) (i.e., L_eq_, equivalent or average sound level during a given period; L_min_, minimum sound level during the measurement period; L_max_, maximum sound level during the measurement period; L_peak_, peak sound level; L_5_, noise level exceeded 5% of the time; and L_95_, the level exceeded for 95% of the time and representing the background level), as well as C-weighted decibel (dBC) sound levels (i.e., L_eq_, L_min_, L_max_, and L_peak_). Sound level meters were set to the A-weighting scale to filter out much of the low-frequency noise (i.e., considered the "normal" limit of human hearing). The C-weighted scale was used to identify impulse noise, defined as “noise consisting of single bursts with a duration of less than one second with peak levels 15 decibels higher than background noise” in 1-minute intervals to estimate exposure to low frequency noise as detailed below [20]. Monitors were factory calibrated prior to use and then pre-calibrated using a Quest QC-10/QC-20 Calibrator (Quest Technologies, Oconomowoc, WI) onsite prior to each measurement. Following each measurement, the monitor was post-calibrated and the data were downloaded using QuestSuite Professional II (Quest Technologies, St. Paul, MN). To protect outdoor monitors, we encased each monitor in an environmental protection kit (3M SoundPro Outdoor Measuring System (SP-OMS), Quest Technologies, Oconomowoc, WI). The University of Maryland, College Park’s Institutional Review Board approved all study protocols. We obtained written informed consent from all study participants prior to any data collection and copies of the consent forms were provided to participants and also retained by the research team.

### Site selection and noise monitoring

We collected 24-hour noise measurements in a total of 11 homes in Doddridge County, WV. Homes were located <300 meters (m) (n = 3 homes); between 300 and 600 m (n = 3 homes), between >600 and 750 m (n = 2 homes), or more than 1000 m (n = 3 homes) from the nearest compressor station. Homes located >1000 m from the nearest compressor station were considered the control homes. Geographic coordinates of the monitor locations were recorded, with sampling locations selected based on convenience and access. None of the homes in our study were located near more than 1 compressor station as verified by visually inspecting a 1000 m radius around each home with Google Earth Pro (version 7.1.7.2602). Because this study was conducted as part of an HIA on UNGDP in the Marcellus Shale in Western Maryland, the minimum proximity distance selected for our study (<300 m) was based on the State of Maryland’s proposed 1,000-foot (304.8 m) setback distance [21] to evaluate whether this distance protects nearby residents from noise levels previously associated with adverse health effects. The setback distance is the minimum distance between natural gas industrial activities and natural or anthropogenic structures [19]. Based on the proximity of our study homes to the nearest compressor station, we generated cutoff points of <300 m (proposed setback distance for the State of Maryland [21]), 300–600 m, >600–750 m, and >1000 m to assess the extent to which increasing the setback distance could potentially reduce noise exposure to nearby residents. Noise monitors were placed inside and outside each home for 24 hours. Indoor monitors were typically placed in a bedroom since it is the room where people spend most of their time when at home, and outdoor monitors were placed in the yard facing the natural gas compressor station. Participants were asked not to play loud music or use the television for 24 hours in the room where the indoor monitor was placed. No other human activity was restricted in the study homes during the monitoring period.

### Data analysis

**Fig 1:**
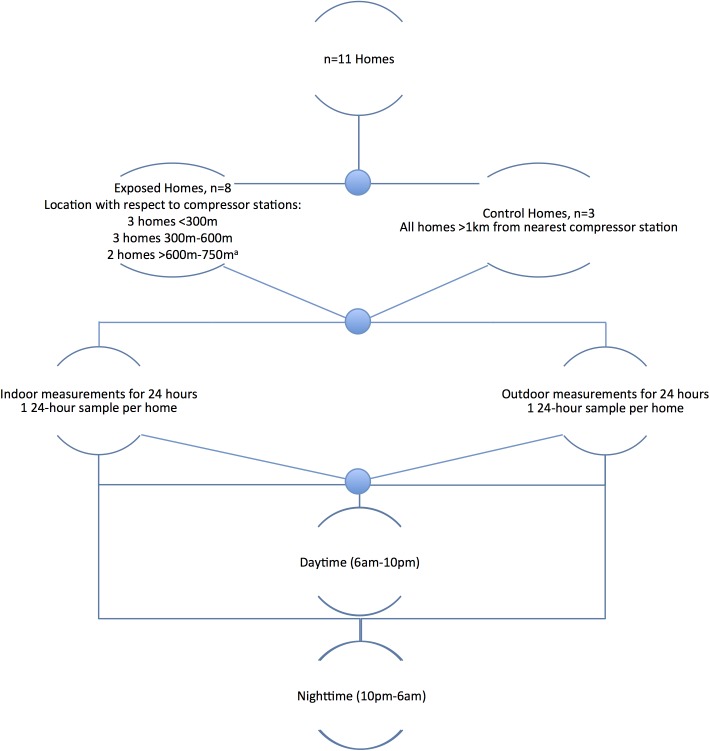
Study design of aggregated noise measurements by exposed homes vs. controls, location (indoors vs. outdoors), and time of day (daytime vs. nighttime).

Summary noise measures were calculated using logarithmic averages and were stratified by distance from the compressor stations (<300 m, 300–600 m, >600–750 m, and >1000 m), time of day (daytime: 7:00 am to 10:00 pm; nighttime: 10:00 pm to 7:00 am), and location (indoors and outdoors), as illustrated in Fig 1. Daytime and nighttime hours were defined as outlined by the U.S. EPA’s “Protective Noise Levels: EPA Levels Document” [17]. The logarithmic averages were calculated utilizing Eq (1) below as described previously by Kheirbek *et al*. [22]:

$$L_{eq,T}=10{log}_{10}\;\left(\frac{1}{N_{t}}\right)\sum 10^{\frac{L_{eq}}{10}}$$ (1)

Where L_eq,T_ is the average equivalent sound level for the time period of interest (T), L_eq_ is the 1-minute interval sound level during the period, and N_t_ is the number of 1-minute interval L_eq_ sound levels taken during the time period of interest [22]. We generated descriptive statistics to assess how A-weighted decibel exposure levels (L_eq,T_) differed based upon select conditions described above (i.e., proximity to compressor stations, indoors versus outdoors, and daytime versus nighttime), and to compare noise levels of the exposed homes (i.e., those located <750 m from the nearest compressor station) to control homes (i.e., those located >1000 m from the nearest compressor station).

We also calculated the combined day-night average sound level or L_dn_ for each home which consists of the A-weighted equivalent sound level for a 24-hour period with a 10 dB penalty to nighttime hours as outlined by the U.S. EPA in the following equation [17]:

$$L_{dn}=10\log\frac{1}{24}\left[[15(10^{\frac{L_{d}}{10}})+9(10^{\frac{L_{n}+10}{10}})]\right]$$ (2)

Where L_d_ is the L_eq_ for daytime hours (7:00 am-10:00 pm) and L_n_ is the L_eq_ for the nighttime hours (10:00 pm-7:00am). The 10 dB nighttime penalty is incorporated in the L_dn_ since noise can be more intrusive at night. For each home, we calculated two L_dn_ values: one consisting of indoor measurements (L_dn, indoors_) and the other consisting of outdoor (L_dn, outdoors_) measurements. These values were generated to compare environmental noise exposure levels observed (i.e., L_dn, indoors_, L_dn, outdoors_) to recommended guidelines set by the U.S. EPA. According to these guidelines, the L_dn_ should not exceed 55 dBA and 45 dBA for outdoor and indoor areas, respectively [17]. We also compared L_eq,T_ values indoors and outdoors during the daytime and nighttime (i.e., L_eq, indoor daytime_, L_eq, indoor nighttime_, L_eq, outdoor daytime_, L_eq, outdoor nighttime_) to guidelines set forth for community noise by the World Health Organization (WHO) [23,24]. WHO guidelines are based on the potential health effects of noise.

Due to growing community concerns for low frequency noise, we also followed the method used by Murphy and King [25] to evaluate the difference between the C-weighted decibel and the A-weighted decibel sound level readings to determine the presence of low-frequency noise. Noise level measurements (dBC and dBA) were aggregated as depicted in Fig 1, via the calculation of logarithmic averages. Subsequently, the difference for each group was calculated. A difference greater than 15 dB indicates a potential for low frequency noise problems and is used as a simple indicator of whether further investigations are necessary.

All statistical analyses were performed using the R Statistical Computing Environment (Version 3.0.2; R Project for Statistical Computing, Vienna, Austria).

## Results

In total, 29,612 one-minute measurements were collected from 11 homes on 22 total sites (11 indoors and 11 outdoors). Six of the homes were trailer homes and five were single-family homes. Environmental noise levels associated with compressor stations were dependent on proximity of residences to the nearest compressor station, sampling location within homes (indoors vs. outdoors), and time of day (daytime vs. nighttime) as outlined below. Additional summary statistics (e.g., L_5_, L_min_, L_max_, L_peak_) by location within the home and distance category are provided as part of the Supplemental Material (S1 Table).

### Noise levels based on proximity of homes to nearest compressor station and location within homes (indoor vs. outdoor)

**Fig 2:**
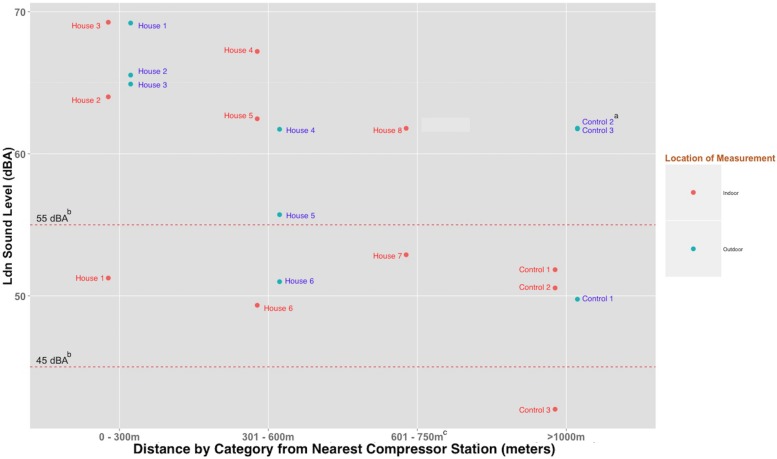
Twenty-four hour A-weighted noise levels indoors and outdoors (L_dn, indoor_, L_dn outdoor_) for each home (dBA) by distance. ^a^Outdoor measurements for Control homes 2 and 3 are similar and thus overlap. ^b^The 55 dBA dashed line denotes the 24-hour EPA recommended limit for outdoor noise level to prevent activity interference and annoyance. The 45 dBA dashed line denotes 24-hour EPA recommended limit for indoor noise level to prevent activity interference and annoyance. ^c^L_dn, outdoor_ for house 7 and 8 is not included in the figure because equipment failure prevented us from measuring for the full 24-hours.

As depicted in Table 1, we found that 24-hour indoor average noise levels (L_eq,24h, indoor_) and combined day-night averages (L_dn, indoor_) were higher for homes located within 750 m of the nearest compressor station compared to control homes (i.e., those located >1000 m from the nearest compressor station) (Table 1, Fig 2). Five out of 6 homes located within 750 m of the nearest compressor station that were monitored for the full 24 hour period had combined day-night indoor average sound levels greater than 60 dBA (Fig 2). For 24-hour outdoor measurements, we found that average noise levels in homes located <300 m from the nearest compressor station were higher than average levels observed in homes located >1000 m from the nearest compressor station (L_eq,24hr, indoor_ = 60.3 dBA vs. 51.6 dBA, respectively). Although we did not observe a clear inverse relationship between 24 hour average outdoor noise levels and proximity of homes to the nearest compressor station (Table 1), we did find that seven of nine homes monitored for the full 24 hours had combined day-night outdoor average sound levels (L_dn, outdoor_) greater than 55 dBA (Fig 2). We also observed that the average outdoor L_95_ (i.e., the sound level exceeded for 95% of the time and representing the background level) for homes located <300 m of the nearest compressor station was 57.5 dBA.

**Table 1:** Summary statistics for 24-hour A-weighted noise levels (dBA) by proximity to nearest compressor station stratified by location within home (indoors vs. outdoors).

	Indoor				Outdoor			
	<300 m	300–600 m	>600–750 m	>1000 m	<300 m	300–600 m	>600–750 m^a^	>1000 m
Number of 1-minute measurements	4320	4320	2880	4384	4320	4320	748	4320
Number of homes	3	3	2	3	3	3	2	3
Mean L_eq,24hr_ (L_dn_)^b^	53.4 (61.0)	53.5 (59.2)	51.2 (57.2)	42.2 (47.9)	60.3 (66.5)	50.3 (56.0)	54.6 (62.3)	51.6 (57.5)
GSD^b^	1.2	1.2	1.1	1.1	1.0	1.1	1.1	1.1
L_95_^c^	45.9	41.8	41.3	37.9	57.5	46.4	48.8	50.2
# homes with L_dn, indoors_ >45 dBA^d^	3/3	3/3	2/2	2/3	—	—	—	—
# homes with L_dn, outdoors_ >55 dBA^e^	**—**	—	—	—	3/3	2/3	N/A^f^	2/3

^a^Number of measurements reduced due to equipment failure; thus sound levels reported include the period sampled which was under 24 hours (i.e., L_eq, T_ not L_eq, 24hr_).

^b^Geometric mean and standard deviation of logarithmic averages for L_eq,24hr_ and L_dn_; GSD for L_eq,24hr_ and L_dn_ were found to be the same.

^c^The level exceeded for 95% of the time and representing the background level.

^d^EPA recommended limit for indoor noise to prevent activity interference and annoyance [17].

^e^EPA recommended limit for outdoor noise to prevent activity interference and annoyance [17].

^f^The # of homes above the EPA recommended limit for outdoor noise is not reported because we were not able to monitor noise levels for an entire 24-hour period due to equipment malfunction.

### Noise levels based on location within homes (indoors vs. outdoors) and time of day (daytime vs. nighttime)

We found that average 24-hour indoor daytime noise levels in homes were higher the closer in proximity they were to compressor stations (Table 2). The geometric mean (GM) for indoor daytime noise levels at the control homes was 43.5 dBA (geometric standard deviation (GSD): 1.2), compared to 56.6 dBA (GSD: 1.3) for homes located within 300 m. The average indoor nighttime noise level in control homes was 41.0 dBA (GSD: 1.0), while the corresponding value for the homes located within 300 m was 50.4 dBA (GSD: 1.1), an increase of 9.4 dBA (Table 2). Similarly, the average outdoor daytime noise level in homes located within 300 m of the compressor station was 61.0 dBA (GSD: 1.0) compared to control home noise levels (GM: 52.7 dBA; GSD: 1.1) (Table 3). The average outdoor noise level at night for homes <300 m from the nearest compressor station was 59.6 dBA (GSD: 1.1), compared to 50.5 dBA (GSD: 1.2) observed outside the control homes during nighttime (Table 3). Outdoor noise levels (L_eq, 24hr, outdoor_) observed indicate that homes located <300 m from a compressor station had the highest noise levels, regardless of when noise levels were monitored (Tables 2 and 3). Although we observed higher average noise levels in homes located <300 m compared to homes located >1000 m from the nearest compressor station, we did not always observe a clear relationship between noise levels and proximity of homes to the nearest compressor station when we stratified by time of day and location (outdoors vs. indoors) within the home. That is, we did not always observe decreasing average noise levels the further away homes were located from the nearest compressor station.

**Table 2:** Summary statistics for A-weighted indoor noise levels (dBA) stratified by distance and time of day.

Time of day home was monitored^a^	Distance to nearest compressor station (meters)	# of homes	# of 1-minute measurements	Mean^b^	GSD^b^	L_95_^b^^,^^c^	# of homes with L_eq, indoors daytime_ >35 dBA^d^	# of homes with L_eq, indoors nighttime_ >30 dBA^e^	L_eq_C-L_eq_A^f^
Day									
	<300	3	2,700	56.6	1.3	44.9	3/3	--	9.0
	300–600	3	2,700	56.1	1.1	42.6	3/3	--	2.3
	>600–750	2	1,800	54.2	N/A^g^	40.2	2/2	--	7.5
	>1,000	3	2,764	43.5	1.2	39.0	3/3	--	10.1
Night									
	<300	3	1,620	50.4	1.1	47.0	--	3/3	16.6
	300–600	3	1,620	51.0	1.3	41.1	--	3/3	4.2
	>600–750	2	1,080	48.3	N/A^g^	42.5	--	2/2	7.7
	>1,000	3	1,620	41.0	1.1	37.0	--	3/3	10.1

^a^Daytime hours are 7:00am-10:00 pm and nighttime hours are 10:00 pm-7:00 am.

^b^Geometric mean of logarithmic averages for L_eq_; GSD = Geometric standard deviation

^c^The level exceeded for 95% of the time and representing the background level.

^d^WHO recommended limit for indoor daytime noise to protect against speech intelligibility and moderate annoyance [23].

^e^WHO recommended limit for indoor nighttime noise to protect against sleep disturbance [23].

^f^Low frequency noise assessment: L_eq_(C)-L_eq_(A) was calculated by finding the logarithmic average for the L_eq_(C) and the L_eq_(A) and then obtaining the difference.

^g^N = 2, therefore a standard deviation was not calculated.

**Table 3:** Summary statistics for A-weighted outdoor noise levels (dBA) stratified by distance and time of day.

Time of day home was monitored^a^	Distance to nearest compressor station (meters)	# of homes	# of 1-minute measurements	Mean^b^	GSD^b^	L_95_^b^^,^^c^	# of homes with L_eq, outdoors daytime_ > 55 dBA^d^	# of homes with L_eq, outdoors nighttime_ >40 dBA^e^	L_eq_C-L_eq_A^f^
Day									
	<300	3	2,700	61.0	1.0	56.9	3/3	—	11.5
	300–600	3	2,700	52.0	1.1	46.3	1/3	—	8.9
	>600–750	2	678^g^	54.2	N/A^h^	47.1	N/A^i^	—	10.5
	>1,000	3	2,700	52.7	1.1	51.1	2/3	—	6.8
Night									
	<300	3	1,620	59.6	1.1	58.2	—	3/3	13.4
	300–600	3	1,620	48.6	1.1	46.6	—	3/3	10.3
	>600–750	2	70^g^	55.4	N/A^h^	52.4	—	N/A^i^	8.0
	>1,000	3	1,620	50.5	1.2	49.3	—	3/3	4.5

^a^Daytime hours are 7:00am-10:00 pm and nighttime hours are 10:00 pm-7:00 am.

^b^Geometric mean of logarithmic averages for L_eq_; GSD = Geometric standard deviation

^c^The level exceeded for 95% of the time and representing the background level.

^d^WHO recommended limit for outdoor daytime noise to protect against moderate annoyance [23].

^e^WHO recommended limit for outdoor nighttime noise to protect against adverse effects such as insomnia, sleep disturbance, sleep quality, and biological effects [24].

^f^Low frequency noise assessment: L_eq_(C)-L_eq_(A) was calculated by finding the logarithmic average for the L_eq_(C) and the L_eq_(A) and then obtaining the difference.

^g^Number of measurements reduced due to equipment failure; thus sound levels reported include the period sampled which was under 24 hours (i.e., L_eq, T_ not L_eq, 24hr_).

^h^N = 2, therefore a standard deviation could not be calculated.

^i^The # of homes above the WHO recommended limit is not reported because we were not able to monitor noise levels for full daytime and nighttime periods due to equipment malfunction.

### Comparison of average noise levels observed to U.S. and WHO guidelines

In our study, we found that average outdoor L_eq, 24h_ and L_dn_ levels for homes <300 m exceeded 55 dBA (i.e., EPA’s recommended limit for outdoor noise levels to prevent activity interference and annoyance) 100% of the time (Table 1). The average outdoor L_dn_ sound levels were also above the EPA’s recommended limit for homes located 300–600 m (GM: 56.0 dBA), as well as control homes (57.5 dBA). We also found that seven of nine homes monitored for the full 24 hours had L_dn, outdoor_ values exceeding 55 dBA (Fig 2). Average indoor levels (L_eq, 24_ and L_dn_) were also above 45 dBA (i.e., EPA’s recommended limit for indoor noise to prevent activity interference and annoyance) for homes located within 750 m from the nearest compressor station. As seen in Fig 2, we found that all but one home (one control home) had indoor combined day-night sound levels (L_dn, indoor_) that exceeded 45 dBA.

We also observed that average L_eq_ levels under different scenarios (e.g., indoors/daytime, outdoors/daytime, etc.) for homes located <300 m of the nearest compressor station exceeded the respective noise levels recommended by the WHO (Tables 2 and 3). In fact, regardless of the proximity of homes to the nearest compressor station, average L_eq_ values observed for indoors (daytime and nighttime), and outdoors (nighttime) exceeded WHO recommendations.

With regards to low frequency noise exposure, we observed a difference between the 24-hour dBA and dBC greater than 15 dB indoors during the night at two homes located <300 m from the nearest compressor station, indicating that residents may be exposed to low frequency noise.

## Discussion

Noise exposure in communities near natural gas compressor stations is a public health concern that has not been adequately addressed. In this pilot study, we found that homes located in close proximity (<300 m) to a compressor station have higher average noise levels, both indoors and outdoors, compared to homes located further away. Residents in these homes could thus potentially be exposed to higher noise levels compared to individuals living in homes located further away. We also found that when examining noise levels for indoors vs. outdoors, a smaller difference existed for homes <300 m relative to the control homes. Additionally, we observed that, in general, daytime noise levels were higher than those observed during the nighttime, and that residents in homes located <300 m from the nearest compressor station may be exposed to low frequency noise. Findings presented herein are from compressor stations in-use and are not related to development activities. As such, they represent chronic noise exposure that community members could potentially experience for years, not transient exposures that cease after the completion of well construction.

In this study, we observed that indoor noise levels (L_eq, 24hr_) for homes located <300 m of a compressor station were on average 6.9 dBA lower compared to outdoors, while for control homes indoor noise levels were 9.4 dBA lower relative to outdoors. The contribution of outdoor noise to indoor noise levels may vary depending on the type of home and whether the windows are opened or closed [17]. For example, based upon recommendations set by the U.S. EPA, a 17 dBA reduction in noise levels would be expected in a cold-climate home with windows open and a 27 dBA reduction with windows closed [17]. In contrast, we only observed a relatively small difference in indoor versus outdoor noise levels at homes near compressor stations. Possible explanations for this small difference include: 1) the homes near the compressor stations were more likely to be trailer homes (6 trailer homes were located within 750 m), while the control homes were single family homes, 2) the indoor noise levels were often higher in the homes <750 m, which would minimize the difference in noise levels, and 3) monitoring took place over the course of one week in April when temperatures ranged from 1 to 21 degrees Celsius, so it is possible that participants had their windows and doors open on the warmer days and closed on cooler days.

In 1974, as a response to the Noise Control Act of 1972, the EPA published a document that outlined noise levels determined to protect public health with an adequate margin of safety. The EPA identified an L_eq, 24hr_ and L_dn_ of 55 dBA for outdoor areas and 45 dBA for indoor residential areas, hospitals, and schools for preventing activity interference and annoyance, and an L_eq, 24hr_ of 70 dBA as the level of environmental noise which will prevent any measurable hearing loss over a lifetime [17]. In 1999, the World Health Organization (WHO) published community noise guidelines, which recommended that outdoor daytime noise levels not exceed 55 dBA and outdoor nighttime noise levels not exceed 45 dBA in order to protect against serious annoyance [23]. Ten years later, the WHO lowered their recommended outdoor nighttime noise level to 40 dBA [24]. In this study, we found that average sound levels routinely exceeded these noise guidelines. However, these comparisons should be interpreted with caution as measurements in this pilot study are for single 24-hr measurements, whereas the limits designated by the U.S. EPA and the WHO represent averages over a period of a year [23,26]. Also, the WHO guidelines provide “values for the onset of health effects from noise exposure” rather than for exposure-response relationships [23]. In addition, some of these guidelines were generated over a decade ago and do not consider the most recent literature on noise exposure and related health effects.

To date, no federal noise standards exist for oil and gas operations; however, several states with active oil and gas development have enacted noise-controlling regulations utilizing a variety of noise standards and/or zoning controls. The noise standards vary by state and generally range from 50 to 60 dBA [27]. To our knowledge, the state of West Virginia does not have any noise standards associated with compressor stations. However, the State of Colorado and the City of Fort Worth in Texas both have noise standards for natural gas compressor stations [28,29]. The noise standards for residential areas in both Colorado and Fort Worth are 55 dBA from 7:00 am to 7:00 pm (daytime) and 50 dBA from 7:00 pm to 7:00 am (nighttime). In Colorado, these standards apply to any well site, production facility, or gas facility with compliance determined by 1-minute measurements collected over a 15 minute sampling period taken at 350 feet from the noise source [29]. In Fort Worth, the measurements are taken at the property line of the receiver [28]. We did not conduct direct comparisons with these standards due to the longer sampling time in our study and the fact that our measurements were not taken at the property line of each home.

With regards to the current proposed setback distance of 300 m for the State of Maryland, our data suggests that this distance may not be sufficient to protect public health based on the average noise levels observed in homes located <300 m from the nearest compressor station and the literature which has reported adverse health effects at noise levels at or below those observed in these homes; however, more studies are needed to confirm this.

While no epidemiological investigations have evaluated health outcomes associated with noise exposures among community members living near natural gas compressor stations, numerous studies have evaluated the health impact of long-term exposure to environmental noise in other contexts (e.g., transportation and urban development). For example, Miedema and Oudshoorn (2001) showed an exposure-response relationship between environmental noise exposure and annoyance level as a result of urban development, including transportation networks and traffic [30]. A growing body of evidence also indicates that exposure to nighttime noise levels as low as 32 dBA can cause a reduction in sleep period, awakenings, sleep stage modifications and autonomic responses, as well as other secondary effects such as inability to concentrate and irritability [9,24,25,31]. In addition, recent research also indicates that nighttime noise exposure to levels greater than 55 dBA may be more relevant for cardiovascular effects than daytime noise exposure [24]. While average indoor nighttime noise levels in our study did not exceed 55 dBA, we did find that average noise levels were greater than 40 dBA; and adverse health effects at or above this level have been reported previously, including sleep disturbance, environmental insomnia, and increased use of somnifacient drugs and sedatives [24]. Studies of noise levels near well pads have also reported higher noise levels during the night, however, we did not observe this in our study [32]. Larger studies are needed to evaluate the effects of time of day on residential noise exposures.

Of importance to consider in future studies is that, in addition to noise-related health outcomes, there may be synergistic effects of noise and air pollution exposure resulting from unconventional natural gas development and production. This is a particular concern for compressor stations that have been found to emit nitrogen oxides (NO_x_), particulate matter (PM), and volatile organic compounds (VOCs) [33,34]. Several studies have evaluated the relationship between air quality and noise on health, but results have been inconsistent [22,35–37]. One study by Huang et al. (2013) found that both air pollution and noise were associated with altered heart rate variability in a study on short-term exposure [37]. Authors reported that noise levels greater than 65.6 dBA seemed to amplify the effects of air pollution when compared with noise levels below this value [37].

To our knowledge, this is the first study to conduct noise monitoring inside and outside of homes located at varying distances from nearby natural gas compressor stations. Our work provides baseline data that can be used to inform future studies. Furthermore, the varying distances of the homes included in our study provide some information on the potential adequacy of current setback regulations. Our study also has some limitations that require acknowledgment. First, the sample size (n = 11 homes) was small with 8 homes located <750 m and 3 homes >1000 m from the nearest compressor station. This was primarily related to a very short deadline the study team had for completing the HIA on the potential public health impacts associated with unconventional natural gas development and production in Maryland [6]. Additionally, participants were recruited through word of mouth by a local community group thus results may not be generalizable; however, they do provide some baseline data to inform larger studies. Also, study homes were selected based on convenience and access. Due to equipment malfunction, outdoor measurements for the two homes located >600–750 m away were not collected for the full 24-hours (daytime period covered 2:30 pm-10:00 pm and the nighttime period covered 10:00 pm-11:09 pm). While these measurements were for only a portion of the 24-hour period, they were still reported as they still provide valuable data for our initial analyses. In addition, other factors that may impact noise levels including topography, weather, wind direction, type of home, and seasonal variation (measurements were collected in early spring over the course of a one-week period) were not captured. Nearby traffic, including traffic count and vehicle type was also not collected, but should be considered in future studies. Other potential noise sources not captured include a major roadway approximately 2 km away from the study homes monitored, occasional heavy truck traffic on the neighborhood road, and natural gas activity along the northern border of the 1000 m radius around one of the compressor stations monitored. However, the L_95_ was found to be similar between day vs. nighttime readings indicating that the background noise level was still high regardless of other potential noise sources. Furthermore, our study only included area-level measurements and did not account for personal noise exposures. Although we searched State public records for permit information on the gas compressor stations in our study, we were not able to get more detailed information such as the volume of gas handled by each compressor that would tell us whether these are “typical” and are representative of compressor noise levels near fracking sites. Finally, choosing appropriate control sites with similar characteristics to the test homes was challenging. The control homes selected for this study were located near a major roadway and also had some local traffic. Selecting controls located in an area with some local traffic potentially introduced additional noise that may not have been found in a completely rural location.

Our study highlights the need for larger noise monitoring studies to be conducted in areas near natural gas development to further assess noise levels, taking into consideration factors such as season, weather, topography, type of home, and to evaluate whether noise levels associated with living in close proximity to compressor stations are associated with adverse health effects to nearby residents. Prolonged exposures to the sound levels observed in this study could increase the risk of adverse health effects to nearby residents. Due to the potential synergistic effects between noise and air pollution, future research should also evaluate both air and noise exposures associated with living near gas compressor stations.

## Conclusion

This pilot study indicates that residents living near a compressor station are potentially exposed to noise levels that are higher than the recommended U.S. EPA levels of 55 dBA (outdoor/daytime) and 45 dBA (indoor/night time). While our results suggest that the currently proposed setback distance by the State of Maryland may not be protective enough, our sample size was small and more research is warranted to determine the exact distance at which future compressor stations should be located to minimize the potential health impacts to nearby residents. States with current UNGDP activities should also consider taking a proactive approach by creating noise and health outcomes surveillance programs to monitor noise levels, as well as the health of residents living in close proximity to natural gas activity.

## Supporting information

S1 Table Additional summary statistics for 24-hour A-weighted noise levels (dBA) by proximity to nearest compressor station stratified by location within home (indoors vs. outdoors). (DOCX)
