# Supplementary material for: A pilot study to assess residential noise exposure near natural gas compressor stations
Source: PLoS One. 2017 Apr 3;12(4):e0174310. doi: 10.1371/journal.pone.0174310 (PMC5378322; doi:10.1371/journal.pone.0174310)
Supplement: S1 Table — (DOCX) [file pone.0174310.s001.docx]

**Table S1.** Additional summary statistics for 24-hour A-weighted noise levels (dBA) by proximity to nearest compressor station stratified by location within home (indoor vs. outdoor).

|  | **Indoor** | | | | **Outdoor** | | | |
| --- | --- | --- | --- | --- | --- | --- | --- | --- |
|  | **<300 m** | **300-600 m** | **>600-750 m^c^** | **>1000 m** | **<300 m** | **300-600 m** | **>600-750 m^c^** | **>1000 m** |
| Number of 1-minute measurements | 4,320 | 4,320 | 2,880 | 4,384 | 4,320 | 4,320 | 748 | 4,320 |
| Number of homes | 3 | 3 | 2 | 3 | 3 | 3 | 2 | 3 |
| L_min_^a^ | 37.5 | 41.4 | 40.8 | 37.5 | 57.3 | 46.1 | 48.3 | 49.9 |
| L_max_^a^ | 62.3 | 63.3 | 64.5 | 50.5 | 67.2 | 56.3 | 64.5 | 55.9 |
| L_5_^a,b^ | 57.6 | 58.5 | 57.7 | 46.7 | 64.8 | 53.3 | 60.5 | 53.6 |
| L_peak_^a,c^ | 84.8 | 83.7 | 86.9 | 70.3 | 83.8 | 76.6 | 86.0 | 74.6 |

^a^Geometric mean of logarithmic averages.

^b^The noise level exceeded 5% of the time.

^c^The peak sound level.

^d^Number of measurements reduced due to equipment failure.
